# Perampanel Attenuates Cognitive and Behavioral Deficits and Modulates Hippocampal Tachykinin Expression in Experimental Temporal Lobe Epilepsy

**DOI:** 10.3390/cimb48100987

**Published:** 2026-09-25

**Authors:** Michaela Shishmanova-Doseva, Darina Barbutska, Yvetta Koeva

**Affiliations:** 1 Department of Pharmacology, Toxicology and Pharmacotherapy, Pharmacy Faculty, Medical University of Plovdiv, 4002 Plovdiv, Bulgaria; 2 Research Institute, Medical University Plovdiv, 4002 Plovdiv, Bulgaria; iveta.koeva@mu-plovdiv.bg; 3 Center for Competence PERIMED-2, Central District, Vasil Aprilov Blvd. 15A, 4002 Plovdiv, Bulgaria; 4 Department of Anatomy, Histology and Embryology, Medical University of Plovdiv, 4002 Plovdiv, Bulgaria

**Keywords:** perampanel, cognitive function, depression, hippocampus, tachykinins

## Abstract

Background: Epilepsy is one of the most prevalent neurological disorders, often presenting alongside many comorbidities. The present study aimed to evaluate the impact of the antiepileptic drug perampanel (PRM) on cognitive function, depressive-like behavior and the expression of substance P (SP) and neurokinin B (NKB) in the hippocampus of naïve rats and rats with an experimental model of temporal lobe epilepsy (TLE). Methods: Vehicle/PRM (3 mg/kg p. os) was administered 3 h after the induction of status epilepticus by lithium–pilocarpine and continued for the next eleven weeks in Wistar rats. Matched naïve rats were treated with the same doses. Results: Long-term treatment with PRM during the chronic phase of TLE improved passive and active learning and memory, as well as spatial working memory, as measured by the Step-down, Shuttle-box avoidance tests and T-maze test. Additionally, the drug alleviated depressive-like behavior in the sugar preference test and hyperactivity in the Activity cage test. In non-epileptic animals, perampanel did not produce significant alterations in the behavioural parameters assessed. Furthermore, we found that TLE animals exhibited the highest levels of immunoreactivity for both tachykinins SP and NKB. However, perampanel treatment attenuated these elevations to varying degrees, depending on the hippocampal region. This was particularly evident in the CA3 subfield and the granular layer of the dentate gyrus. In non-epileptic animals, perampanel produced only minor changes relative to vehicle-treated controls. In conclusion, the observed cognitive improvement and antidepressant-like behavior associated with perampanel are presumed to result from its impact on the hippocampal tachykinin expression.

## 1. Introduction

Epilepsy is one of the most prevalent neurological disorders, with more than 60 million people living with it worldwide [1]. It is characterized by a persistent predisposition to epileptic seizures, which result from abnormal, excessive and synchronous electrical activity affecting different regions of the brain [2]. The modern definition encompasses not only recurrent unprovoked seizures, but also their associated neurobiological, cognitive, psychological and social consequences.

Epilepsy can affect people of all ages, genders and social status. Around 5 million new cases are reported each year [3]. The main causes are head injuries, infections, strokes and genetic factors. However, in around half of all cases, the exact cause remains unknown [1]. Furthermore, only 70% of patients can achieve a seizure-free state with proper treatment.

The clinical manifestation of the disease varies depending on the location and spread of pathological electrical activity. Some patients also experience accompanying symptoms such as sensory disturbances, automatisms, or behavioral changes [4]. Therefore, epilepsy can lead to a number of complications and long-term consequences [5,6]. Cognitive decline is a significant aspect of the clinical presentation of epilepsy and can affect various cognitive domains, including memory, attention, executive functions, language, and processing speed [7]. The extent of these impairments varies widely and depends on numerous factors, such as the disease’s cause, age of onset, seizure frequency and type, and the location of the epileptogenic focus [6].

Psychiatric comorbidities, such as depression and anxiety, are much more prevalent among people with epilepsy than in the general population [8]. They often significantly impact on the quality of life, social integration and professional fulfilment for those affected. Depressive symptoms are a significant part of the overall disease burden as they often remain undiagnosed and untreated for extended periods [9,10]. Depressive disorders in people with epilepsy are characterized by a range of symptoms, including low mood, anhedonia, fatigue, sleep disturbances, appetite changes, a sense of hopelessness and low self-esteem. Suicidal thoughts and behavior are also exhibited by some patients [9]. Therefore, early recognition and appropriate treatment are essential. In this regard, some antiepileptic medications (AEMs) can be beneficial in reducing seizure activity and associate depressive symptoms in patients with epilepsy.

Perampanel is a non-competitive, selective inhibitor of AMPA glutamate receptors, which have a pivotal function in rapid excitatory synaptic transmission within the central nervous system. By inhibiting these receptors, the drug reduces pathological neuronal excitability and suppresses the spread of epileptic activity [11,12]. The mechanism of action of perampanel is unique among antiepileptic drugs, as it modulates postsynaptic glutamatergic transmission without directly affecting sodium or calcium channels. This makes it an effective treatment for patients with refractory forms of epilepsy for whom other therapies have been unsuccessful [13]. The clinical effects of perampanel include reducing seizure frequency, particularly in cases of focal seizures with or without secondary generalisation and primary generalised tonic–clonic seizures. Randomised clinical trials demonstrate a significant reduction in seizures when perampanel is added to standard therapy [13]. In terms of safety, the most commonly reported adverse reactions are drowsiness, dizziness, irritability and behavioural changes. Psychiatric symptoms such as aggression and anxiety have also been reported in some patients [14,15].

Neurokinin B (NKB) and substance P (SP) are increasingly recognized as important modulators of neuronal excitability, synaptic transmission, cognition, and emotional behavior. They belong to the tachykinin family of neuropeptides and exert their biological effects through G-protein-coupled neurokinin receptors [16]. SP preferentially activates the neurokinin-1 receptor (NK1R), whereas NKB is the endogenous ligand with the highest affinity for the neurokinin-3 receptor (NK3R) [17,18]. Both tachykinins signaling systems are widely represented within the central nervous system and have been implicated in the modulation of neuronal activity and behavior [17,19,20]. In particular, growing evidence suggests that NK3R signaling is involved in a range of neuronal functions beyond its well-established neuroendocrine role, including the modulation of neuronal excitability and cognitive processes [18].

SP/NK1R signaling has been associated with stress-related responses [21] and psychiatric disorders, such as depression and anxiety [22]. This provides a potential connection between tachykinin signaling and the psychiatric manifestations frequently accompanying neurological disorders [16,19]. SP is particularly relevant to epilepsy, as accumulating experimental evidence suggests a proconvulsant role of SP/NK1R signaling. Alterations in SP expression and release have been observed following seizure activity, while activation of the SP/NK1R system has been associated with increased seizure susceptibility in experimental models [23,24]. Moreover, a landmark experimental study demonstrated increased hippocampal expression of the SP precursor during status epilepticus showing that SP facilitates hippocampal glutamate release. These findings suggest a functional interaction between tachykinin and excitatory glutamatergic signaling [25].

Considering the central role of glutamate-mediated excitation in epilepsy and the mechanism of perampanel as a selective non-competitive AMPA receptor antagonist, changes in hippocampal tachykinin signaling may provide additional information about neurochemical mechanisms accompanying its anticonvulsant, cognitive and behavioral effects. Evaluation of SP and NKB expressions may therefore help characterize the involvement of neuropeptidergic modulation in the hippocampal response to perampanel treatment. In this context, the present study aimed to evaluate the impact of the antiepileptic drug perampanel on cognitive functions, depressive-like behavior, and the hippocampal expression of substance P and neurokinin B in an experimental model of temporal lobe epilepsy (TLE).

## 2. Materials and Methods

### 2.1. Drugs and Reagents

Lithium chloride (LiCl), Scopolamine methyl nitrate and Pilocarpine hydrochloride were purchased from Sigma Aldrich (Aldrich Chemie GmbH, Hamburg, Germany). Diazepam (Sopharma, Sofia, Bulgaria); Perampanel (Eisai Ltd., Hatfield, Hertfordshire, UK); Bouin’s Fluid Sigma, HT10132; Gill’s Hematoxylin Solution, No. 2, sc-24973A (Santa Cruz Biotechnology Inc., Santa Cruz, CA, USA); ImmunoCruz goat ABC Staining, sc-2023 (Santa Cruz Biotechnology Inc., Santa Cruz, CA, USA); Substance P (NC1/34HL), sc-21715 and Neurokinin B (1B2), sc-517127 (Santa Cruz Biotechnology Inc., Santa Cruz, CA, USA); VectaMount^®^ Express Mounting Medium, H-5700-60 (Vector Laboratories, Newark, CA, USA).

### 2.2. Animals

All experiments were performed in strict accordance with the guidelines of the European Community Council directives 86/609/EEC. 0.2010/63/EC. Permission for the use of animals in experiments No. 206/01.10.2018 was obtained from the Bulgarian Food Safety Agency, as well as from the Ethical Committee on Human and Animal Experimentation at Medical University of Plovdiv No. 1/28.02.2019.

Male Wistar rats weighing 180–200 g (n = 32) were obtained from the Animal Center of Medical University of Plovdiv. The animals were housed under standard laboratory conditions: a 12:12-h light–dark cycle, relative humidity of 45%, an ambient temperature of 23 ± 1 °C, and free access to food and water.

### 2.3. Induction of Chronic State of Temporal Lobe Epilepsy (TLE) with Lithium–pilocarpine and Group Allocation

Half of the rats were injected with 127 mg/kg of lithium chloride (LiCl) intraperitoneally (i.p.) 24 h before receiving a low dose of 30 mg/kg of pilocarpine (i.p.). Thirty minutes prior to the pilocarpine injection, 1 mg/kg of scopolamine methyl nitrate was administered intraperitoneally (i.p.) to neutralize the peripheral cholinergic effects. Following the final injection, the animals were placed in separate cages and their seizure activity was assessed using the Racine scale [26] as previously described [27,28]. The criterion for reaching stage 4/5 of SE was sustained motor seizures (defined as more than nine seizures per hour) for at least two hours. Only animals that met this criterion were used in subsequent experiments. Following two hours of continuous convulsions, 10 mg/kg of diazepam was administered i.p. This was followed by repeated administration of the same dosage as required. Approximately three hours after the Li-Pilo injections, the rats were given a subcutaneous injection of an equal volume of a solution of 0.9% NaCl and 5% glucose (up to 3% of body weight) to restore volume and reduce mortality. Treatment with Veh or perampanel (PRM), administered orally via gavage, commenced three hours after the onset of SE and continued for up to eleven weeks. From the beginning of the ninth week all rats were subjected to a series of tests (Figure 1).

The rats were randomly divided into four groups (n = 8) as follows:

○ Group 1 (C-veh) treated with saline p. os.
○ Group 2 (C-TLE) treated with saline p. os and with induced TLE with Li-Pilo i.p.
○ Group 3 (C-PRM) treated with perampanel 3 mg/kg p.os.
○ Group 4 (TLE-PRM) treated with perampanel 3 mg/kg p.os and with induced TLE with Li-Pilo i.p.

### 2.4. Behavioural Tests

#### 2.4.1. Activity Cage

The spontaneous locomotor activity of the rats was measured using an automated cage fitted with photo-sensors (Activity Cage, Ugo Basile, Gemonio, Italy). The number of horizontal and vertical movements made by each animal was recorded automatically for a period of five minutes.

#### 2.4.2. T Maze Test

The rats’ spatial memory was assessed using a place-learning test in a T-maze based on spatial orientation and food reward. To ensure motivation, rats from all experimental groups underwent the same 24-h food deprivation procedure before T-maze testing, with water available ad libitum throughout. This procedure was applied uniformly across groups to standardize motivation for obtaining the food reward, thereby minimizing between-group differences in reward-driven task performance. Each animal underwent a training session consisting of a total of 11 trials, comprising one initial forced trial and 10 subsequent choice trials. During the forced trial, one arm of the maze was closed off while food pellets were placed in the other arm to encourage the animal to enter it. In the subsequent ten choice trials, both arms were fully accessible, with the food reward remaining in the same arm. In each trial, the animal was placed at the base of the T-shaped maze, and the arm it chose was recorded. The inter-trial interval was five minutes. Cognitive capacity was quantified using a spatial memory index, calculated by dividing the number of correct choices by the total number of trials (n = 10).

#### 2.4.3. Two-Way Active Avoidance Test with Negative Reinforcement

A test for active and passive avoidance (Shuttle box, Ugo Basile, Gemonio, Italy) was used to assess active and passive learning and memory. The learning session was held for five consecutive days and consisted of 30 trials per day. Each trial consisted of 6 s of light and sound stimulation (670 Hz and 70 dB), followed by 0.4 mA of foot electrical stimulation through the grid floor within 3 s. There was a 12-s rest period after each session. On the 7th day after the last training, a retention test for long-term memory traces was performed. The behavioural parameters measured were the number of avoidances (i.e., the number of correct responses to conditioned stimuli), the number of escapes (i.e., the number of unconditioned responses), and the number of inter-trial crossings.

#### 2.4.4. One-Way Step-Down Passive Avoidance Test with Negative Reinforcement

A step-down device (Ugo Basile, Gemonio, Italy) was used to assess passive learning and memory. Following the standard programme for this apparatus, all rats underwent three training sessions, each lasting 60 s, with an interval of 60 min between each session. Each rat was placed on a plastic platform that vibrated vertically when the apparatus was switched on. If a rat stepped off the platform with three or four paws, an electrical stimulus (0.4 mA) was delivered through the slatted floor of the cage for three seconds. The animal is considered trained if it remains on the platform for more than 60 s in two consecutive training sessions. The long-term memory test uses the same protocol as the training sessions but without electrical stimulation. It is conducted 24 h after the final training session.

#### 2.4.5. Sugar Preference Test for Depressive-like Behavior

The sugar preference of single-housed rats was assessed over three days using a two-bottle choice test. The test is conducted over three consecutive days. On the first day, two water bottles are placed in front of each rat. On the second day (the pre-test), one bottle is filled with water and the other with a sugar solution. On the third day, the actual test is conducted with two 100 mL water bottles provided, one filled with water and the other with a 1% sugar solution. After 24 h, the amount consumed from each bottle is recorded. A lack of preference for the sugar solution indicates depressive-like behavior.

The investigators performing the behavioural tests were not blinded to the experimental groups during the collection or analysis of the data.

At the end of the experiment, all animals were decapitated under deep anesthesia, which was induced with 0.2 mg/kg fentanyl and 0.3 mg/kg midazolam administered intraperitoneally. The brains were removed immediately and collected for further investigation.

### 2.5. Immunohistochemistry

Hippocampal tissue samples (5 μm) were processed according to standard histological procedures, embedded in paraffin, sectioned, and mounted on silane-coated glass slides. The sections were deparaffinized in xylene and rehydrated through a graded series of ethanol solutions. Endogenous peroxidase activity was blocked by incubation with methanol containing hydrogen peroxide for 30 min. The sections were subsequently washed in phosphate-buffered saline (PBS) for 15 min and incubated with normal swine serum diluted in PBS for 30 min to reduce nonspecific binding.

Immunohistochemical detection of substance P and neurokinin B was performed using the avidin–biotin complex (ABC) method with the ImmunoCruz™ ABC Staining System and 3,3′-diaminobenzidine (DAB) as the chromogen. Sections were incubated with monoclonal primary antibodies against Substance P (clone NC1/34HL, sc-21715; monoclonal IgG) and Neurokinin B (clone 1B2, sc-517127; monoclonal IgG1κ) (both from Santa Cruz Biotechnology, Inc., USA), diluted 1:100, for 24 h at 4 °C in a humidified chamber. Negative control sections were processed in parallel, with PBS substituted for the primary antibody.

Following completion of the immunohistochemical reaction, the sections were rinsed with PBS, counterstained with hematoxylin, dehydrated through a graded series of ethanol solutions, and coverslipped using VectaMount mounting medium.

Representative microphotographs of SP and NKB immunoreactivity were obtained using a Leica DM3000 microscope equipped with a FLEXACAM C3 digital camera (Leica Microsystems).

### 2.6. Quantitative Analysis of Immunohistochemical Staining Intensity

Quantitative morphometric analysis of SP and NKB immunoreactivity was performed separately using the Olympus DP-Soft image analysis system (version 4.1 for Windows) equipped with a Camedia C-5050ZOOM digital camera (Olympus, Hachioji, Tokyo, Japan).

The intensity of DAB staining was quantified using a grayscale range from 0 to 256, where 0 represented white and 256 represented black; thus, higher values corresponded to greater staining intensity. Specific immunoreactivity was identified as dark brown to black staining within positively labeled cells. To ensure comparability between experimental groups, tissue sections from all groups were processed and analyzed in parallel under identical experimental conditions.

For each animal, 20 hippocampal sections from both hemispheres were analyzed at ×400 magnification. Multiple microscopic fields were examined, and staining intensity was determined in at least 100 immunopositive cells per section. Mean staining intensity was calculated for each animal and used for subsequent statistical analyses. SP and NKB immunoreactivity were expressed in relative units (RUs).

For the quantitative immunohistochemical analysis, the slides were coded prior to evaluation and the investigator performing the measurements was unaware of the experimental group assignment. The group identity was only revealed after the quantitative analysis was complete.

### 2.7. Statistical Analysis

The analysis was conducted using IBM SPSS^®^ statistical software, version 19.0. The results were analyzed using parametric tests because of normally distributed data, as assessed by the Kolmogorov–Smirnov test. The two-way active avoidance test was performed several times during the experiment, and the results were analyzed using a one-way ANOVA for repeated measures with time as the within-subject factor. The main effect of time was examined as well as its size (η). Mauchly’s test of sphericity was applied to examine whether the assumption of sphericity was met (*p* > 0.05) or violated (*p* < 0.05). In the second case to adjust degrees of freedom of numerator and denominator the Greenhouse–Geisser adjustment factor was applied. The results of the other behavioural tests (activity cage, T-maze, step-down test and sugar preference test), as well as hippocampal expression of substance P and neurokinin B, were analyzed using a one-way ANOVA. When the F-ratio was significant, the between-group differences were assessed by Tukey’s or Games Howell post hoc tests depending on the homogeneity of the dispersion (as determined using a Levene’s test). A *p* value of less than 0.05 (*p* < 0.05) was considered statistically significant.

## 3. Results

### 3.1. Activity Cage

Examining motor activity, ANOVA revealed a significant effect of perampanel treatment [F(3,31) = 7.661, *p* < 0.001]. Between-group analysis showed that the epilepsy model group exhibited a significant increase in horizontal movements compared to both the control group (*p* = 0.003) and the TLE-PRM group (*p* = 0.021) (Figure 2). However, no difference was detected in the number of vertical movements [F(3,31) = 0.765, *p* = 0.524].

### 3.2. T Maze Test

ANOVA revealed a significant impact of perampanel treatment [F(3,31) = 5.888, *p* = 0.003] on spatial working memory. Rats with the TLE model notably decreased their working memory index compared to control animals (*p* = 0.004), while the TLE-PRM group significantly increased their index compared to the epilepsy model group (*p* = 0.017) (Figure 3).

### 3.3. Two-Way Active Avoidance Test with Negative Reinforcement

According to the one-way repeated measures ANOVA, Mauchly’s test revealed that the assumption of sphericity had been violated χ^2^ (14) = 35.54, *p* < 0.001. Therefore, degrees of freedom were corrected using Greenhouse–Geisser estimates of sphericity. The analysis revealed a significant main effect of time [F(3.45, 106.86) = 36.95, *p* < 0.001, η = 0.544]. Analysis of variance revealed a treatment effect on the number of conditioned responses only on days 3 [F(3,28) = 3.375, *p* = 0.032], 4 [F(3,28) = 6.583, *p* = 0.002] and 5 [F(3,28) = 23.081, *p* < 0.001], as well as during the retention test on day 12 [F(3,28) = 11.622, *p* < 0.001]. The *post-hoc* test showed a notable decrease in the number of avoidances by the TLE-C group on days 3 (*p* = 0.018), 4 (*p* = 0.008) and 5 (*p* < 0.001), as well as on day 12 (*p* = 0.001), compared to the control animals (Figure 4). The number of conditioned responses significantly increased in rats with a TLE model and treated with PRM on days 4 (*p* = 0.003), 5 (*p* < 0.001) and 12 (*p* < 0.001) compared to the epilepsy model group (Figure 4).

One-way ANOVA for repeated measures demonstrated that Mauchly’s test of sphericity assumption had been violated χ^2^ (14) = 39.34, *p* < 0.001, therefore degrees of freedom were corrected using Greenhouse–Geisser estimates of sphericity. The analysis revealed no significant main effect of the within-subject factor time [F(3.26, 101.11) = 1.82, *p* = 0.144, η = 0.055]. ANOVA demonstrated a significant treatment-related effect on the number of unconditioned responses during all days of the learning session 1 [F(3,28) = 4.937, *p* = 0.007], 3 [F(3,28) = 3.591, *p* = 0.026], 4 [F(3,28) = 10.896, *p* < 0.001] and 5 [F(3,28) = 8.090, *p* < 0.001], and during the memory retention test [F(3,28) = 10.129, *p* < 0.001]. The group with a model of TLE notably decreased the number of escapes on days 1 (*p* = 0.016), 3 (*p* = 0.033), 4 (*p* < 0.001), 5 (*p* = 0.001) and 12 (*p* < 0.001) compared to the control group (Figure 5). A significant increase in the number of unconditioned responses was demonstrated by the TLE-PRM group on days 4 (*p* < 0.001), 5 (*p* = 0.003) and 12 (*p* = 0.002) in comparison with the animals with a TLE model (Figure 5).

Analysis of variance revealed no significant main effect of the within-subject factor time [F(5, 155) = 1.09, *p* = 0.364, η = 0.034]. During the first three days of the learning session, ANOVA did not detect any significant treatment-related effects on the number of inter-trial crossings. However, a notable impact was detected on day 4 [F(3,28) = 3.878, *p* = 0.019] and during the retention test [F(3,28) = 5.433, *p* = 0.005]. The between group analysis revealed that the TLE group led to a significant increase in the number of the inter-trial crossings on days 4 (*p* = 0.032) and 12 (*p* = 0.009) compared to control rats (Figure 6). The TLE-PRM group notably decreased their number during the retention test on day 12 (*p* = 0.011), compared to the epilepsy group.

### 3.4. One-Way Step-Down Passive Avoidance Test with Negative Reinforcement

ANOVA showed a significant treatment-related impact during the learning session of the passive avoidance step-down test [F(3,28) = 4.863, *p* = 0.008]. Post-hoc test revealed that the TLE group spent less time on the platform than the control rats (*p* = 0.006) and the TLE-PRM group (*p* = 0.039) (Figure 7).

The same effect was observed in the memory retention test [F(3,28) = 8.260, *p* < 0.001]. Between-group differences showed that the epilepsy group had a shorter passive latency time in comparison with the control animals (*p* < 0.001) and the epilepsy group treated with PRM (*p* = 0.004) (Figure 7).

### 3.5. Sugar Preference Test (SPT) for Depressive-like Behavior

For the SPT the analysis of variance demonstrated a notable impact of the treatment [F(3,28) = 19.088, *p* < 0.001]. The post hoc test showed that the epilepsy group exhibited depressive-like behaviour compared to the control group (*p* < 0.001), while PRM treatment during epileptogenesis only partially reversed it (*p* = 0.008, TLE-PRM compared the TLE-C group; *p* = 0.012, TLE-PRM compared the C-veh group) (Figure 8).

### 3.6. Neurokinin-B Hippocampal Expression

ANOVA revealed a significant effect of treatment on neurokinin-B (NKB) expression in the different hippocampal subfields: CA1 [F(3,24) = 12.143, *p* < 0.001], CA2 [F(3,24) = 11.894, *p* < 0.001], CA3 [F(3,24) = 76.292, *p* < 0.001] and the Gr-DG [F(3,24) = 9.807, *p* < 0.001]. Post hoc test demonstrated that the TLE-C group significantly increased the expression of the peptide in the CA1, CA2 and CA3 subfields (*p* < 0.001, respectively) as well as in the Gr-DG (*p* = 0.001) compared to the control animals (Figure 9). Treatment with PRM during epileptogenesis only partially decreased neurokinin-B expression in the CA3 subfield (*p* < 0.001) and Gr-DG (*p* = 0.043) compared to the TLE-C group, while no effect was observed in the CA1 and CA2 subfields (*p* > 0.05). Moreover, the TLE-PRM group showed higher expression of the peptide in several subregions of the hippocampus: CA1 (*p* = 0.045), CA2 (*p* = 0.008) and CA3 (*p* < 0.001), compared to the control rats. A between-group analysis revealed higher neurokinin-B expression in the TLE-PRM group compared to the C-PRM group in the CA1 (*p* = 0.018), CA2 (*p* = 0.005) and CA3 (*p* < 0.001) subfields (Figure 9). However, no difference was detected between the control animals and the C-PRM group (*p* > 0.05).

### 3.7. Neurokinin-B Immunohistochemical Expression

Immunohistochemical localization of Neurokinin-B in the dorsal hippocampus (Figure 10A–D) in the cornu ammonis (CA1, CA2, and CA3) subfields, including in the dentate gyrus, and granular cell layer (GrDg).

### 3.8. Substance P Hippocampal Expression

The analysis of variance revealed a notable impact of treatment on Substance P expression in the hippocampal subregions: CA1 [F(3,24) = 14.313, *p* < 0.001], CA2 [F(3,24) = 16.722, *p* < 0.001], CA3 [F(3,24) = 67.060, *p* < 0.001] and the Gr-DG [F(3,24) = 57.123, *p* < 0.001]. A between-group analysis showed that the epilepsy group increased the expression of the peptide in all subfields CA1, CA2, CA3 and Gr-DG (*p* < 0.001, respectively) compared to the matched control group (Figure 11). The TLE-PRM animals partially alleviated the epilepsy-induced Substance P expression in some of the subfields of the hippocampus CA1 (*p* = 0.041), CA3 (*p* = 0.007) and Gr-DG (*p* = 0.001) in comparison with the C-TLE group. Furthermore, the TLE-PRM group exhibited higher peptide expression in CA1 (*p* = 0.031), CA2 (*p* = 0.002) CA3 (*p* < 0.001) subfields and the Gr-DG (*p* < 0.001) compared to the matched C-PRM rats (Figure 11). No difference was detected between the control animals and the C-PRM group (*p* > 0.05).

### 3.9. Substance P Immunohistochemical Expression

Immunohistochemical localization of Substance P in the dorsal hippocampus (Figure 12A–D) in cornu ammonis (CA1, CA2, and CA3) subfields, as well as in the granular cell layer in the dentate gyrus (GrDG).

## 4. Discussion

The current study found that epileptic animals exhibited increased spontaneous locomotor activity and long-term neurobehavioral deficits due to epileptogenesis. It is well known that this process triggers permanent pathological changes to the hippocampal–limbic neural networks during the chronic stage of the model. These include neuronal loss, reactive gliosis, synaptic remodeling, and persistent glutamatergic hyperexcitability [29]. In contrast to the effects on horizontal activity, perampanel did not significantly impact vertical activity. As vertical activity in the Activity Cage reflects rearing behaviour, these differential effects on horizontal and vertical movements suggest that perampanel does not suppress all components of spontaneous locomotor activity uniformly. However, given the relatively short five-minute observation period and the absence of dedicated assessments of motor coordination and sedation, these findings cannot rule out the possibility that the reduction in horizontal activity observed was due to the sedative or other behavioural effects of perampanel.

The two-way active avoidance test with negative reinforcement revealed that rats with lithium–pilocarpine-induced epilepsy displayed significant impairments in active learning and memory. They exhibited a notably lower number of conditioned and unconditioned responses compared to the naïve controls. Treatment with perampanel substantially improved cognitive performance, presented with significant increase in conditioned responses in both learning and memory sessions. These results suggest that perampanel has a beneficial effect on active learning and memory processes.

An important observation is that administering perampanel to naïve animals did not result in any statistically significant changes to the number of conditioned and unconditioned responses compared to naïve controls. These data demonstrate that, under physiological conditions, perampanel does not adversely affect cognitive function, while simultaneously showing potential to reverse learning and memory impairments induced by chronic epilepsy.

Furthermore, a similar effect was observed in the passive avoidance test, in which we found that perampanel improved learning and formation of long-term memory traces in epileptic animals. Moreover, the drug facilitated the formation of hippocampal-dependent spatial working memory. The consistent results from these behavioural tests, which assess different forms of associative learning and memory, provide compelling evidence of perampanel’s beneficial effect on cognitive impairments induced by chronic epilepsy. Notably, in naïve animals, perampanel did not lead to any deterioration in cognitive performance, suggesting that the drug does not impair learning and memory processes under physiological conditions.

Improvements in learning and memory in animals treated with perampanel are likely due to its beneficial effect on the neural networks involved in memory formation and consolidation. The hippocampus plays a central role in these processes, being responsible for forming new memories, associative learning and long-term synaptic plasticity [30]. In chronic temporal lobe epilepsy, the hippocampus is one of the structures most severely affected, with a loss of pyramidal neurons observed in the CA1 and CA3 regions, damage to hilar neurons in the dentate gyrus, reactive gliosis, a proliferation of mossy fibres and abnormalities in long-term potentiation (LTP) processes [30,31,32]. These structural and functional changes disrupt the hippocampal neural network and are considered a major cause of the cognitive impairments that are characteristic of chronic temporal lobe epilepsy. In addition to the hippocampus, the amygdala, which processes the emotional component of learning, and the prefrontal cortex, which ensures the integration of memory processes and executive control, are involved in the formation of aversive memory [33]. Consequently, the improved performance observed in active and passive avoidance tests in our current study may indicate the restoration of function in the hippocampal–limbic neural circuit.

Our results are consistent with those of other experiments, which found that perampanel improves cognitive function and significantly suppresses seizure activity via different mechanisms of action. In addition to its potent anticonvulsant properties, in pilocarpine-induced status epilepticus, treatment with perampanel has been shown to mitigate impairments in learning and memory when evaluated using the Y-maze and Novel Object Recognition tests. The authors also report that the drug reduced caspase-3 activation in the hippocampus, suggesting a reduction in apoptotic processes and a potential neuroprotective effect [34]. In an experimental study of rats with chronic epilepsy, Kim et al. demonstrated that the antiepileptic effect of perampanel is closely linked to modulation of the PTEN/PI3K/Akt signaling pathway. Treatment with perampanel restores PTEN expression, suppresses the pathologically activated PI3K/Akt signaling pathway and reduces AMPA-receptor-mediated neuronal hyperexcitability in the hippocampus [35]. The authors suggest that these molecular changes contribute to the suppression of epileptic activity and the preservation of normal function in hippocampal neural networks. This may also be significant for improving cognitive impairment associated with chronic epilepsy [35]. Furthermore, in an experimental PTZ-kindling model, treatment with perampanel was found to raise the seizure threshold and lead to significant improvements in behavioural abnormalities [36]. The effect of perampanel was associated with normalizing the expression of the BDNF/TrkB signaling pathway and reducing the expression of pro-inflammatory markers in brain tissue [36]. Another experimental study using a PTZ-kindling model of epilepsy found that perampanel significantly suppressed epileptic activity, improved cognitive function and reduced anxiety-like behavior by alleviating oxidative stress and neuroinflammation [37].

The present study found that treatment with perampanel increased the consumption of a sugar solution by animals with experimental epilepsy. This result suggests a reduction in anhedonia and a beneficial effect on depressive-like behaviour. The etiology of depression in epilepsy is multifactorial [10]. Biological mechanisms include dysfunction of neurotransmitter systems, particularly the serotonergic and noradrenergic systems, as well as structural and functional changes in brain regions such as the limbic system and prefrontal cortex [6,38,39]. Although the evidence regarding the effect of perampanel on affective disturbances in epilepsy is inconclusive, the results of the present study are consistent with experimental data obtained using the WAG/Rij model, in which perampanel exhibits both anticonvulsant and antidepressive-like properties. This lends weight to the idea that the drug may affect common pathophysiological mechanisms underlying both epilepsy and associated depression [40].

The observed improvements in cognition and behaviour were accompanied by alterations in hippocampal tachykinin immunoreactivity, suggesting a potential connection between these effects and tachykinin signaling. A broadly similar pattern of SP and NKB immunoreactivity was observed across the investigated hippocampal subfields. TLE animals exhibited the highest levels of immunoreactivity for both tachykinins, whereas perampanel treatment attenuated these increases to varying degrees depending on the hippocampal region. In non-epileptic animals, perampanel produced only minor changes relative to vehicle-treated controls. Overall, these findings suggest that enhanced hippocampal tachykinin signaling accompanies the epileptic state.

The increase in SP immunoreactivity observed in TLE animals is consistent with experimental evidence linking the SP/NK1R system to seizure-related changes in neuronal excitability [23,24]. SP expression has been shown to increase markedly in hippocampal principal neurons during status epilepticus, including neurons of the CA1 and CA3 and the dentate gyrus, while SP itself can facilitate glutamate release from hippocampal tissue [25]. Furthermore, the experimental manipulation of SP/NK1R signaling has demonstrated that this pathway can influence both the seizure susceptibility and severity [23,25]. Along these lines, the intrahippocampal administration of SP has been reported to promote seizure activity, whereas NK1R antagonism reduces seizure severity in several experimental paradigms [23]. Taken together, these findings support the hypothesis that increased SP signaling may participate in a positive feedback mechanism that sustains or amplifies hippocampal hyperexcitability during prolonged epileptic activity [23,25].

Notably, the pattern of NKB immunoreactivity was broadly comparable to that of SP. However, in contrast to SP, the involvement of the NKB/NK3R signaling system in epilepsy has received considerably less attention. Nevertheless, earlier experimental evidence has demonstrated pronounced long-lasting alterations in hippocampal NKB expression following limbic seizures, including increased NKB immunoreactivity and mRNA expression in distinct hippocampal neuronal populations [41]. NKB is the high-affinity endogenous ligand of NK3R, which is widely expressed throughout the nervous system. It has been implicated in the regulation of neuronal excitability, as well as processes related to learning, memory, and affective behavior [18]. Therefore, the increased hippocampal NKB immunoreactivity observed in TLE animals may reflect epilepsy-associated remodeling of tachykinin signaling within hippocampal networks. Nevertheless, as direct evidence of a causal proconvulsant role for NKB/NK3R signaling in TLE is limited, the present findings should be interpreted as evidence of an association with the epileptic state, rather than as proof of direct epileptogenic action by NKB.

Furthermore, the hippocampus represents an important site at which NK3R signaling may influence cognitive processes. NK3R is expressed in hippocampal regions involved in memory formation, including the CA1, CA3 and the dentate gyrus [42]. Experimental modulation of NK3R alters memory consolidation, supporting a functional role of the NKB/NK3R system in learning and memory processes. In mice, the pharmacological blockade of NK3R produced sex-dependent alterations in recognition memory and NK3R expression was demonstrated throughout the dorsal hippocampal circuitry [42]. These findings indicate that alterations in NKB/NK3R signaling may contribute to changes in hippocampal-dependent cognitive functions.

The parallel changes in SP and NKB are noteworthy and may indicate a broader involvement of the tachykinin system in hippocampal responses to epilepsy. Although SP and NKB preferentially activate different neurokinin receptors and their functional roles are not identical, both peptides can modulate neuronal signaling within the CNS [43]. Their concomitant increase in the epileptic hippocampus may therefore represent a coordinated component of neuropeptidergic plasticity occurring in response to sustained neuronal hyperactivity. Whether these changes contribute to seizure generation and propagation or instead represent a compensatory response to recurrent seizures cannot be determined from the present findings and warrants further investigation.

Taken together, the present findings indicate that experimental TLE is accompanied by coordinated alterations in hippocampal SP and NKB signaling, supporting a broader involvement of tachykinin-mediated neuropeptide plasticity in the neurochemical reorganization associated with chronic epilepsy. Further studies targeting individual neurokinin receptors are warranted to clarify the functional significance of these alterations and their potential contribution to epileptic network dysfunction and cognitive impairments.

The study’s main limitations include the absence of continuous EEG monitoring throughout the 11-week treatment period. This prevented the quantification of chronic-phase seizure burden, as well as the isolation of direct perampanel effects from seizure reduction. Furthermore, the use of only male Wistar rats limits the generalisability of the findings to females. Evaluating a single perampanel dose does not permit the assessment of dose-dependent effects on behavioural outcomes or hippocampal SP or NKB immunoreactivity. Therefore, further research incorporating EEG monitoring, both sexes, and multiple doses is required to validate these mechanisms and their translational relevance.

## 5. Conclusions

In conclusion, the present study demonstrates that long-term perampanel treatment during the chronic phase of experimental temporal lobe epilepsy resulted in a notable reduction in hyperactivity and an improvement in various cognitive domains, including passive and active learning and memory, as well as spatial working memory. Furthermore, the antiepileptic drug mitigated the accompanying depressive-like behavior. These beneficial impacts could be linked to a decrease in tachykinin signaling, as perampanel treatment reduces increased neurokinin B and substance P immunoreactivity in the hippocampus. These findings may represent an important mechanism contributing to the beneficial effects of perampanel and suggest that modulation of hippocampal tachykinin signaling may be involved in the neurochemical adaptations associated with experimental temporal lobe epilepsy.

## Figures and Tables

**Figure 1:**
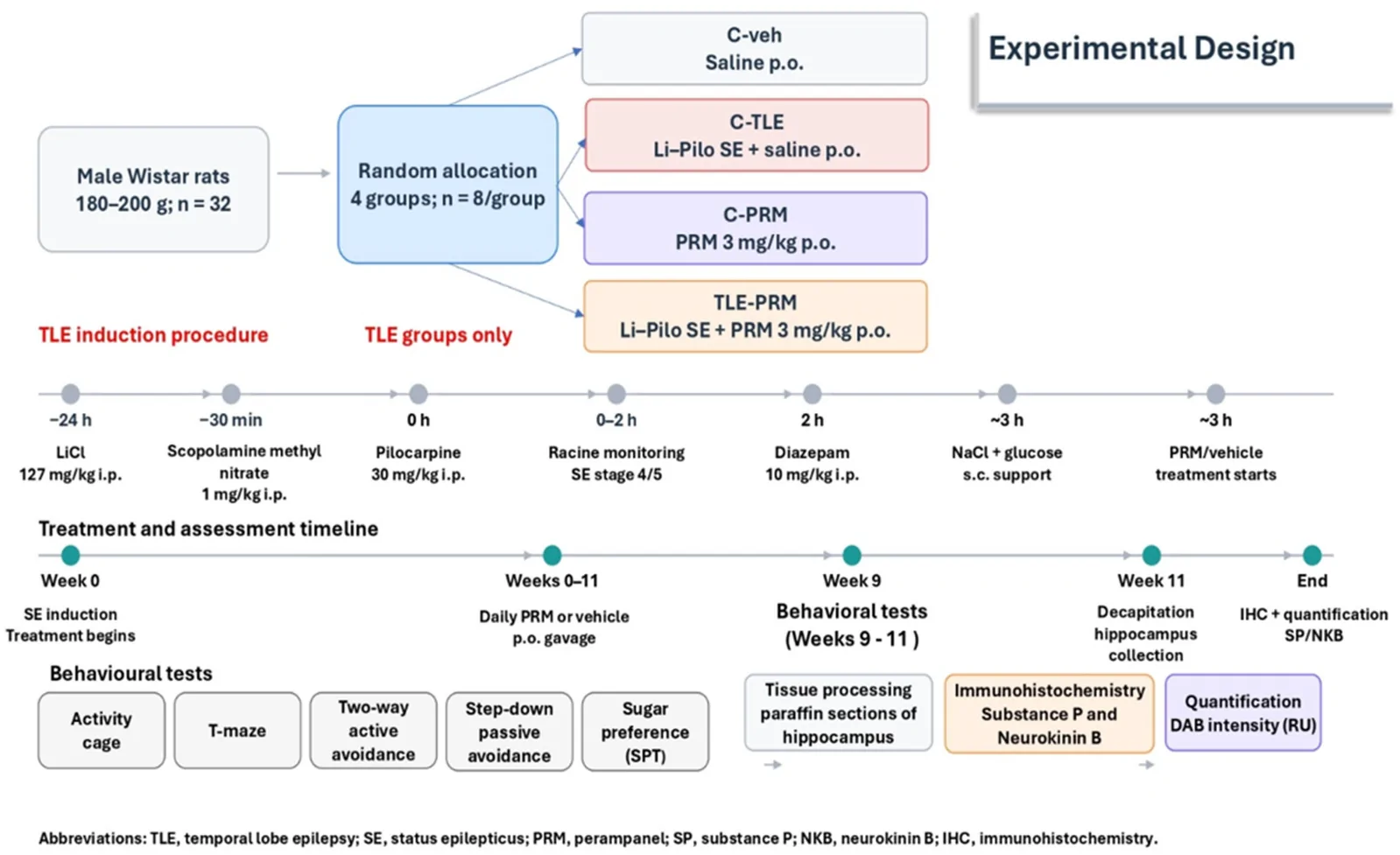
Experimental design.

**Figure 2:**
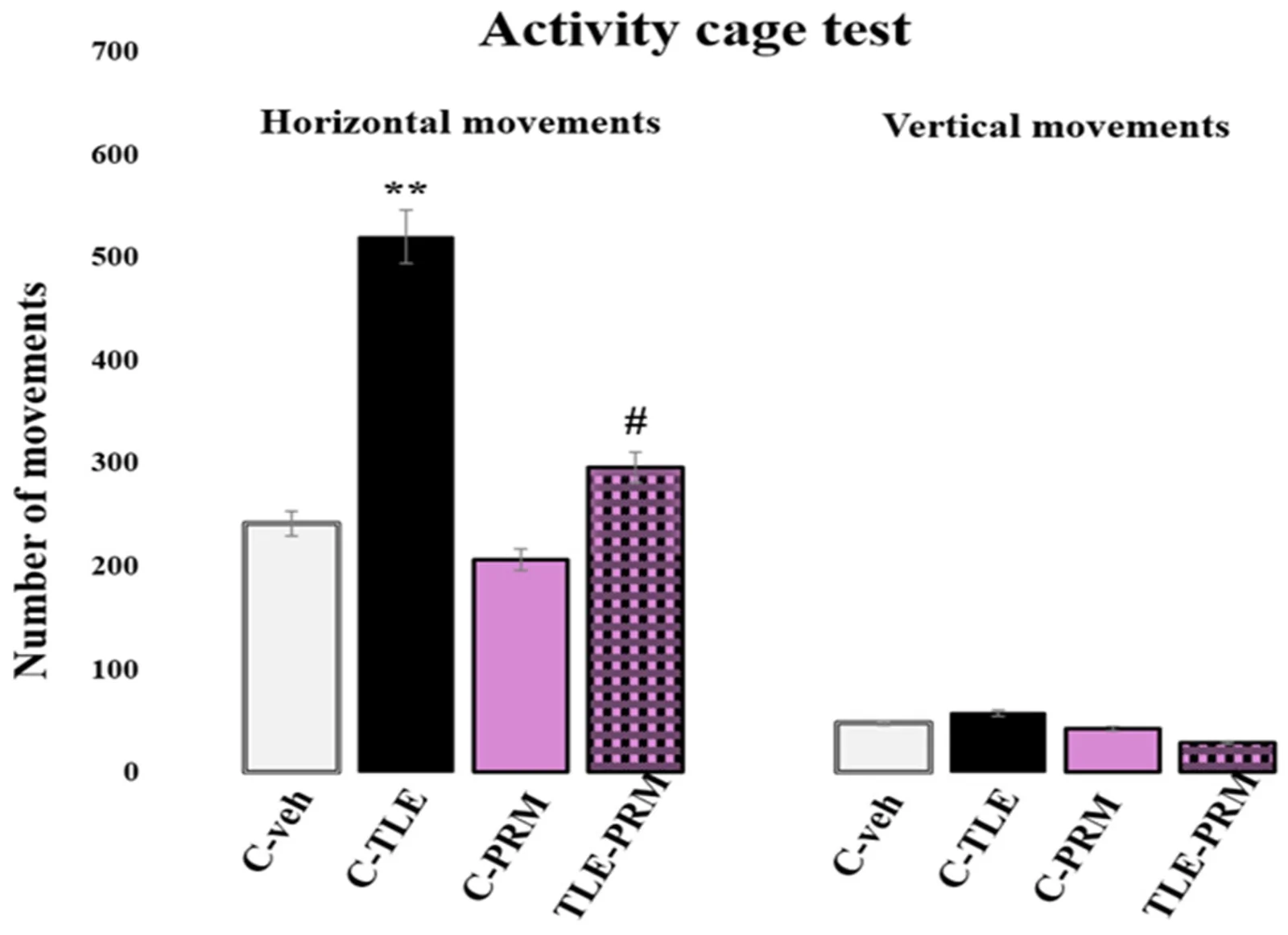
Effect on the number of horizontal and vertical movements of rats with lithium–pilocarpine-induced temporal lobe epilepsy (TLE) and following long-term perampanel (PRM) treatment in the outlined experimental groups: vehicle-treated controls (C-veh), rats with temporal lobe epilepsy (C-TLE), perampanel-treated controls (C-PRM), and perampanel-treated rats with temporal lobe epilepsy (TLE-PRM). Data are presented as mean ± SEM. ** *p* < 0.01 vs. C-veh; ^#^ *p* < 0.05 vs. C-TLE.

**Figure 3:**
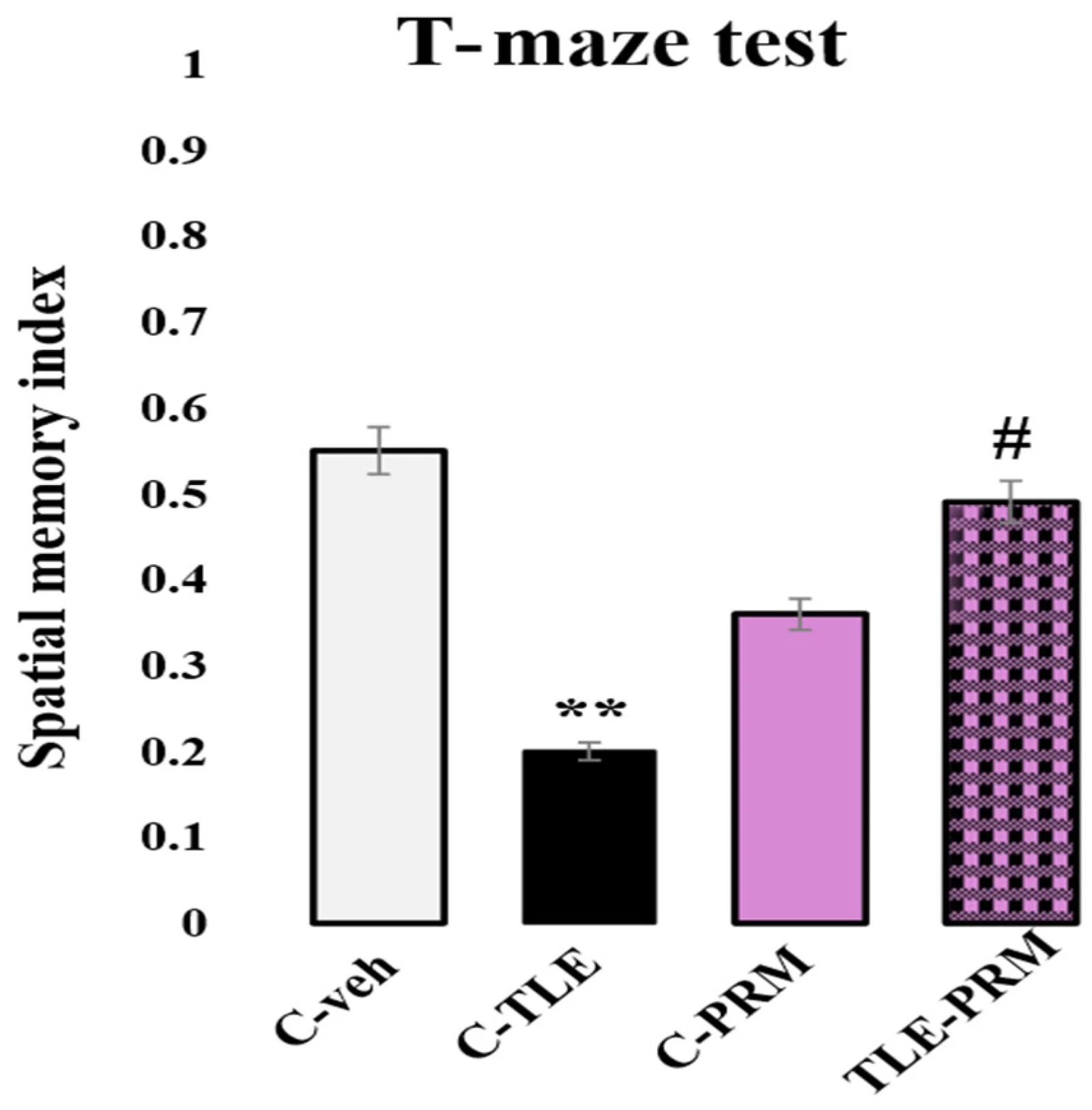
Effect on the spatial memory index of rats with lithium–pilocarpine-induced temporal lobe epilepsy (TLE) and following long-term perampanel (PRM) treatment in the outlined experimental groups: vehicle-treated controls (C-veh), rats with temporal lobe epilepsy (C-TLE), perampanel-treated controls (C-PRM), and perampanel-treated rats with temporal lobe epilepsy (TLE-PRM). Data are presented as mean ± SEM. ** *p* < 0.01 vs. C-veh; ^#^ *p* < 0.05 vs. C-TLE.

**Figure 4:**
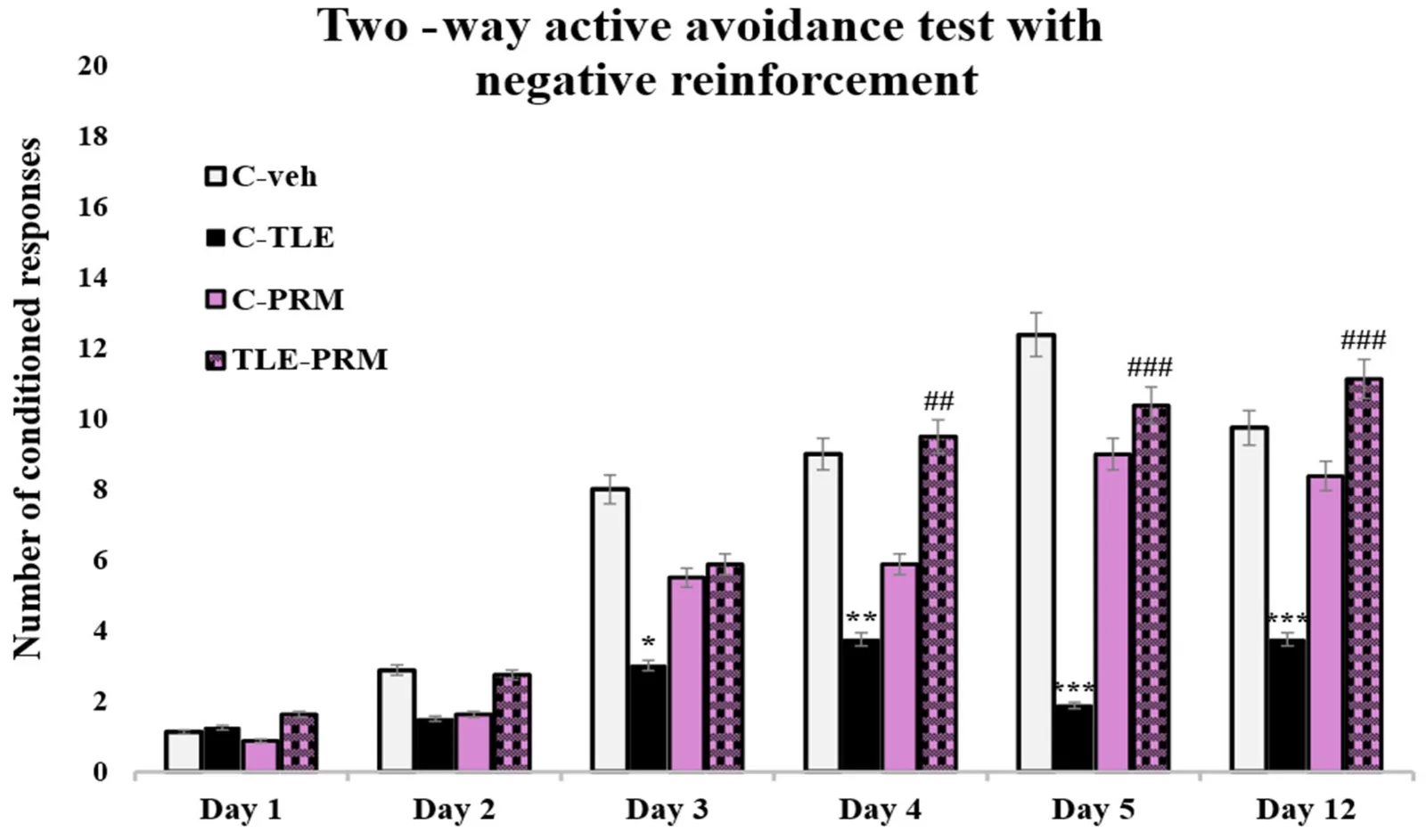
Effect on the number of conditioned responses of rats with lithium–pilocarpine-induced temporal lobe epilepsy (TLE) and following long-term perampanel (PRM) treatment in the outlined experimental groups: vehicle-treated controls (C-veh), rats with temporal lobe epilepsy (C-TLE), perampanel-treated controls (C-PRM), and perampanel-treated rats with temporal lobe epilepsy (TLE-PRM). Data are presented as mean ± SEM. * *p* < 0.05, ** *p* < 0.01, *** *p* < 0.001 vs. C-veh; ^##^ *p* < 0.01, ^###^ *p* < 0.001 vs. C-TLE.

**Figure 5:**
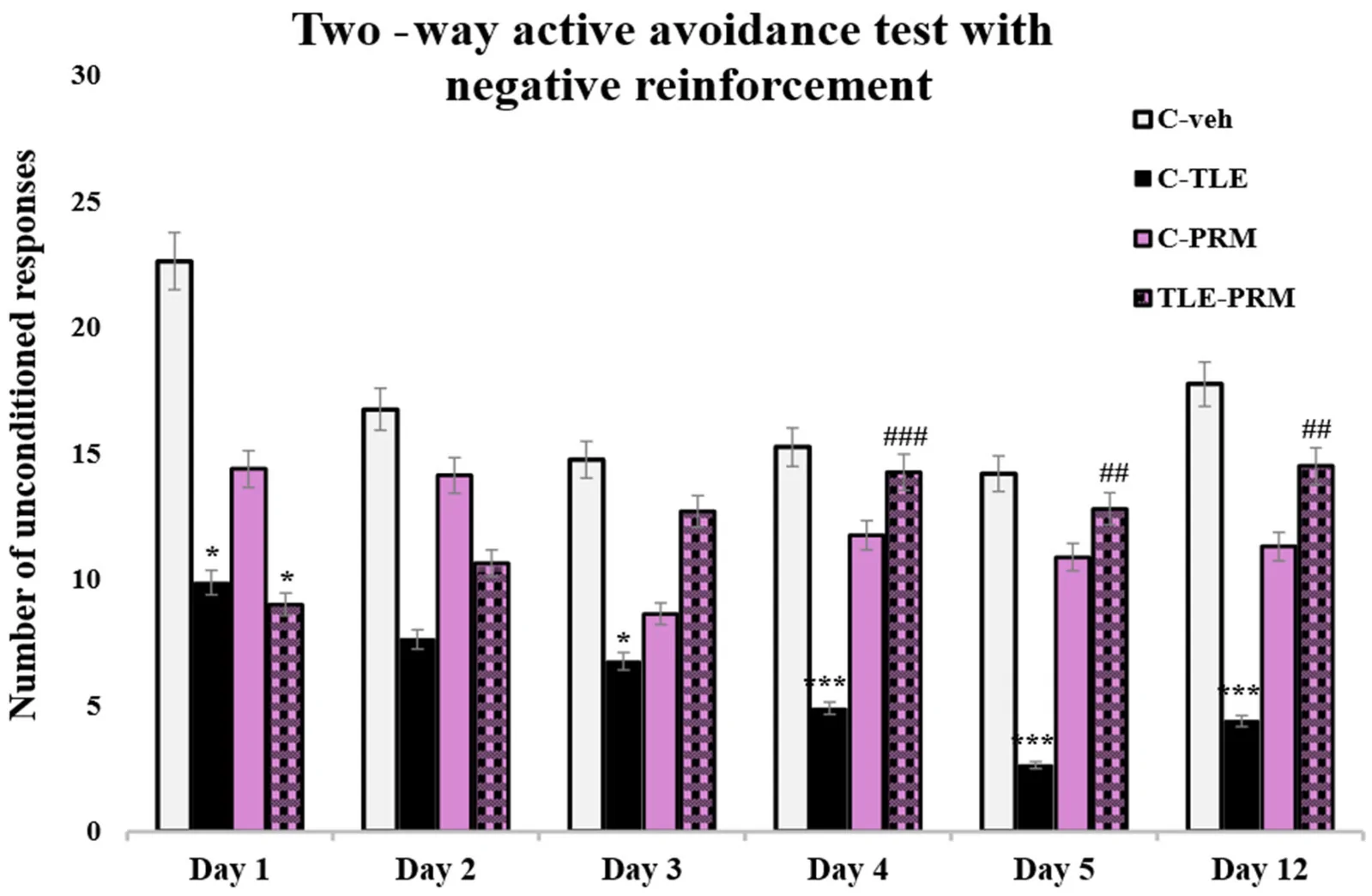
Effect on the number of unconditioned responses of rats with lithium–pilocarpine-induced temporal lobe epilepsy (TLE) and following long-term perampanel (PRM) treatment in the outlined experimental groups: vehicle-treated controls (C-veh), rats with temporal lobe epilepsy (C-TLE), perampanel-treated controls (C-PRM), and perampanel-treated rats with temporal lobe epilepsy (TLE-PRM). Data are presented as mean ± SEM. * *p* < 0.05, *** *p* < 0.001 vs. C-veh; ^##^ *p* < 0.01, ^###^ *p* < 0.001 vs. C-TLE.

**Figure 6:**
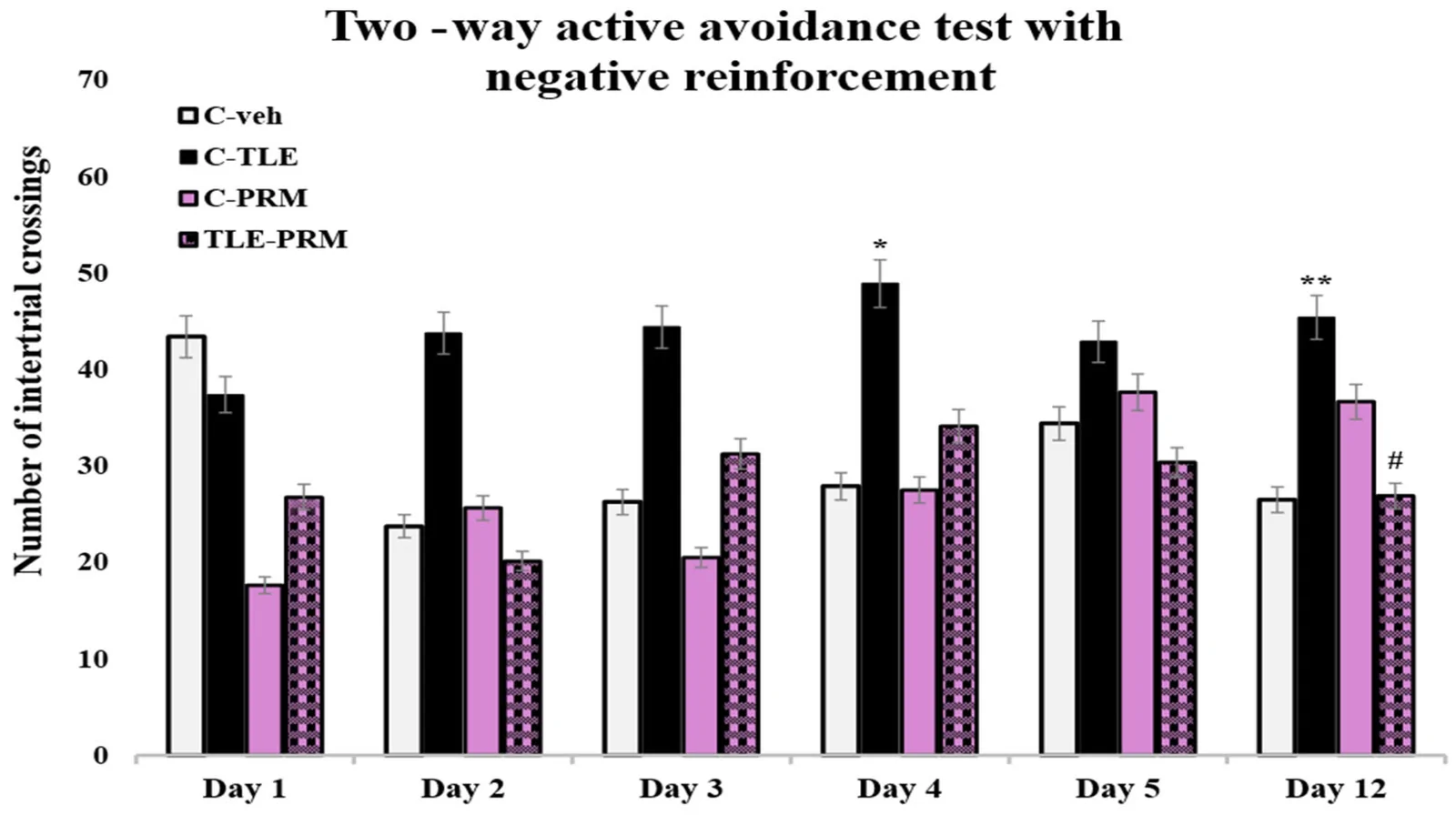
Effect on the number of inter-trial crossings of rats with lithium–pilocarpine-induced temporal lobe epilepsy (TLE) and following long-term perampanel (PRM) treatment in the outlined experimental groups: vehicle-treated controls (C-veh), rats with temporal lobe epilepsy (C-TLE), perampanel-treated controls (C-PRM), and perampanel-treated rats with temporal lobe epilepsy (TLE-PRM). Data are presented as mean ± SEM. * *p* < 0.05, ** *p* < 0.01 vs. C-veh; ^#^ *p* < 0.05 vs. C-TLE.

**Figure 7:**
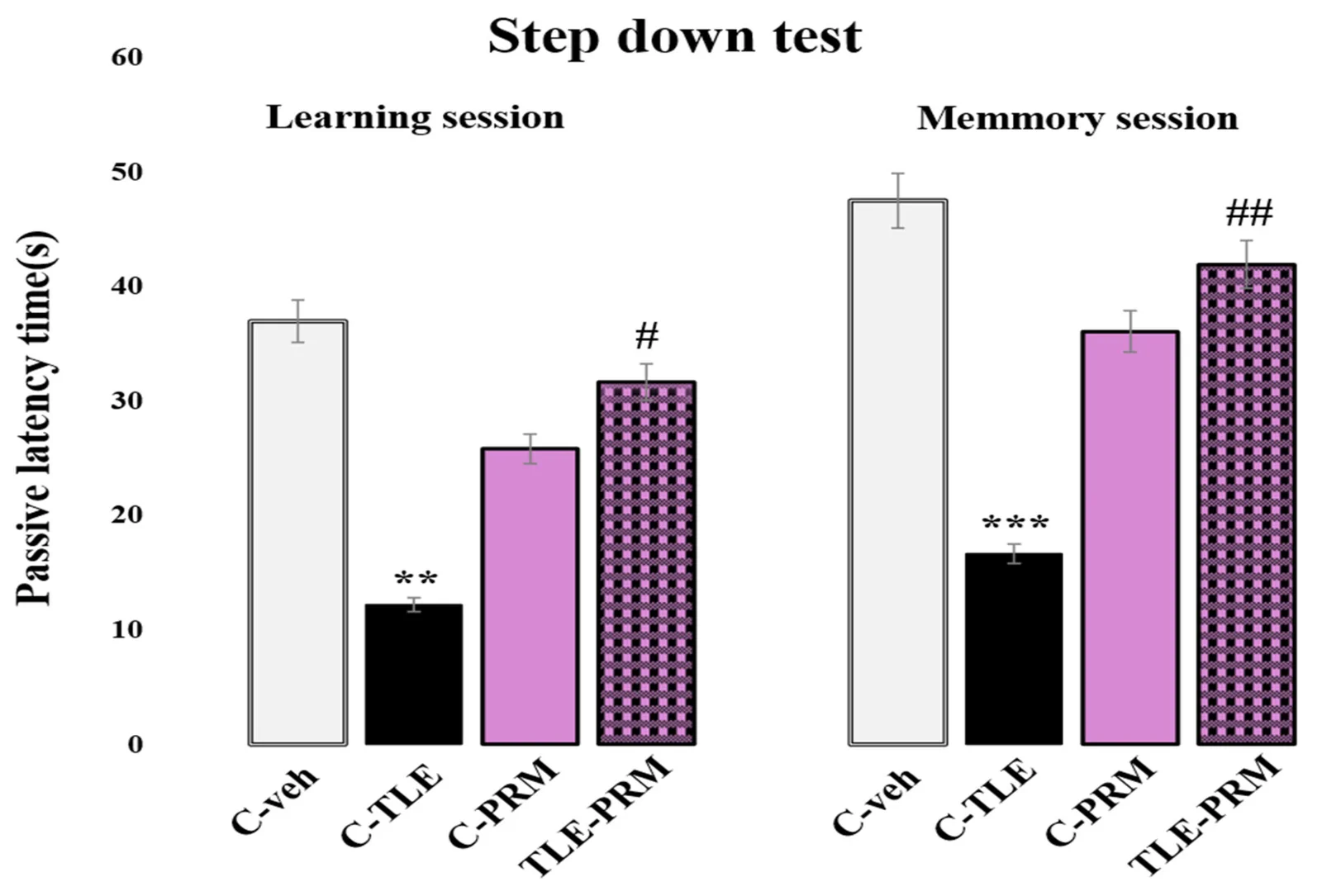
Effect on the passive latency time (s) of rats with lithium–pilocarpine-induced temporal lobe epilepsy (TLE) and following long-term perampanel (PRM) treatment in the outlined experimental groups: vehicle-treated controls (C-veh), rats with temporal lobe epilepsy (C-TLE), perampanel-treated controls (C-PRM), and perampanel-treated rats with temporal lobe epilepsy (TLE-PRM). Data are presented as mean ± SEM. ** *p* < 0.01, *** *p* < 0.001 vs. C-veh; ^#^ *p* < 0.05, ^##^ *p* < 0.01 vs. C-TLE.

**Figure 8:**
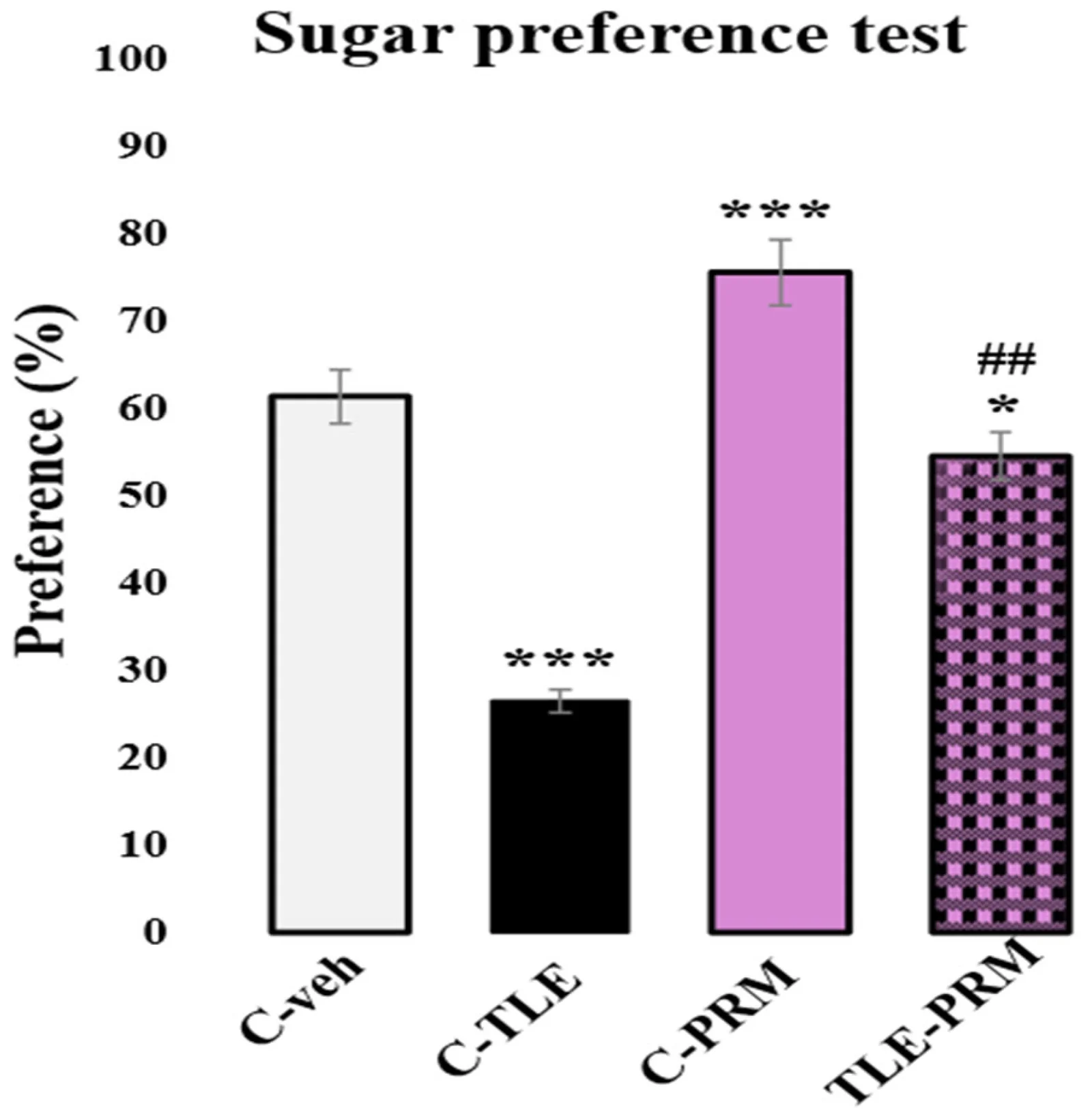
Effect on sugar consumption of rats with lithium–pilocarpine-induced temporal lobe epilepsy and following perampanel (PRM) treatment in the outlined experimental groups: vehicle-treated controls (C-veh), rats with temporal lobe epilepsy (C-TLE), perampanel-treated controls (C-PRM), and perampanel-treated rats with temporal lobe epilepsy (TLE-PRM). Data are presented as mean ± SEM. * *p* < 0.05, *** *p* < 0.001 vs. C-veh; ^##^ *p* < 0.01 vs. C-TLE.

**Figure 9:**
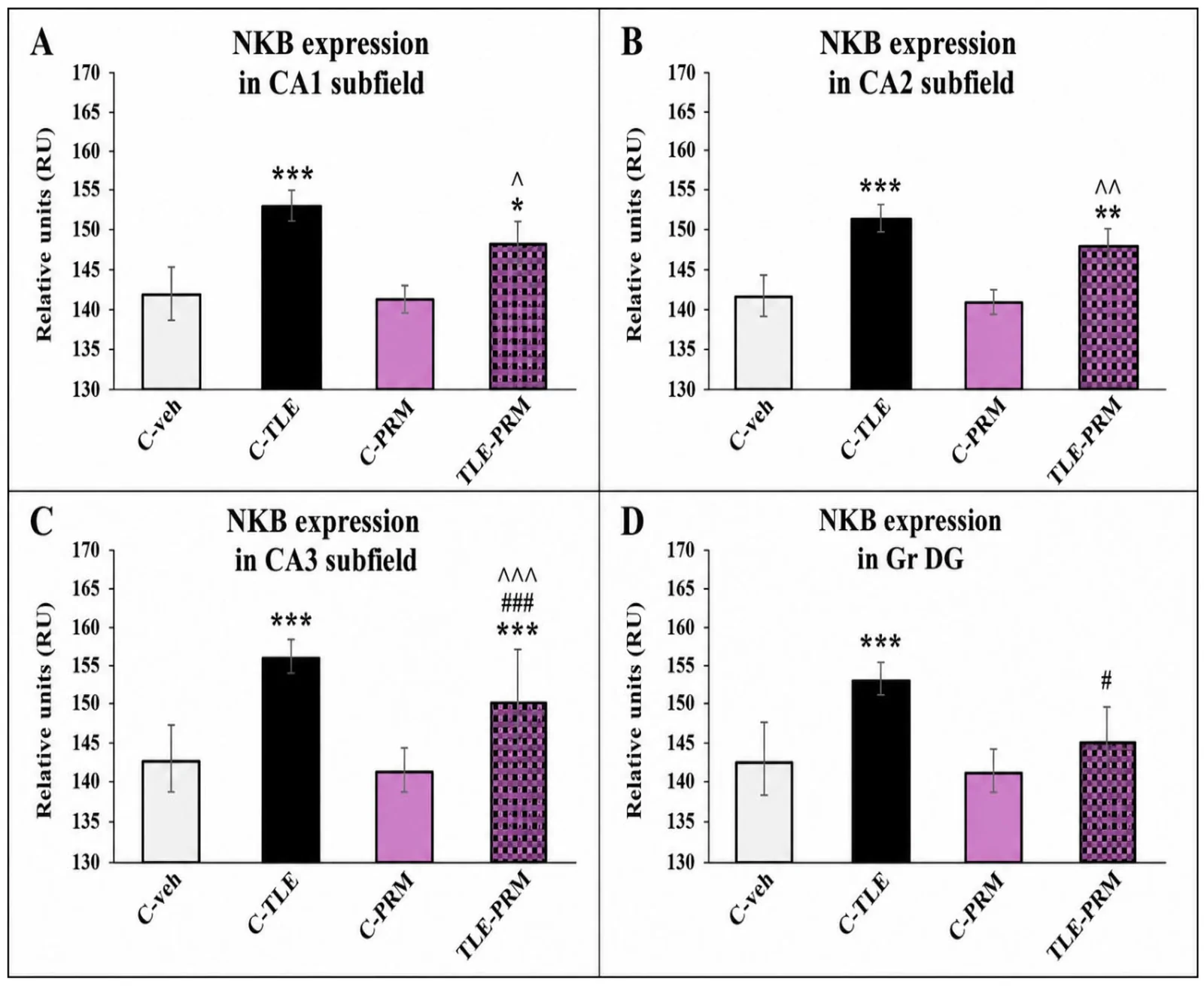
Neurokinin-B (NKB) expression in the hippocampus of rats with lithium–pilocarpine-induced temporal lobe epilepsy and following perampanel (PRM) treatment. Quantitative analysis of NKB immunoreactivity in the CA1 (**A**), CA2 (**B**), CA3 (**C**), and granular layer of the dentate gyrus (GrDG) (**D**). NKB expression is presented as relative units (RUs) in the following experimental groups: vehicle-treated controls (C-veh), rats with temporal lobe epilepsy (C-TLE), perampanel-treated controls (C-PRM), and perampanel-treated rats with temporal lobe epilepsy (TLE-PRM). Data are presented as mean ± SEM. * *p* < 0.05, ** *p* < 0.01, *** *p* < 0.001 vs. C-veh; ^#^ *p* < 0.05, ^###^ *p* < 0.001 vs. C-TLE; ^ *p* < 0.05, ^^ *p* < 0.01, ^^^ *p* < 0.001 vs. C-PRM.

**Figure 10:**
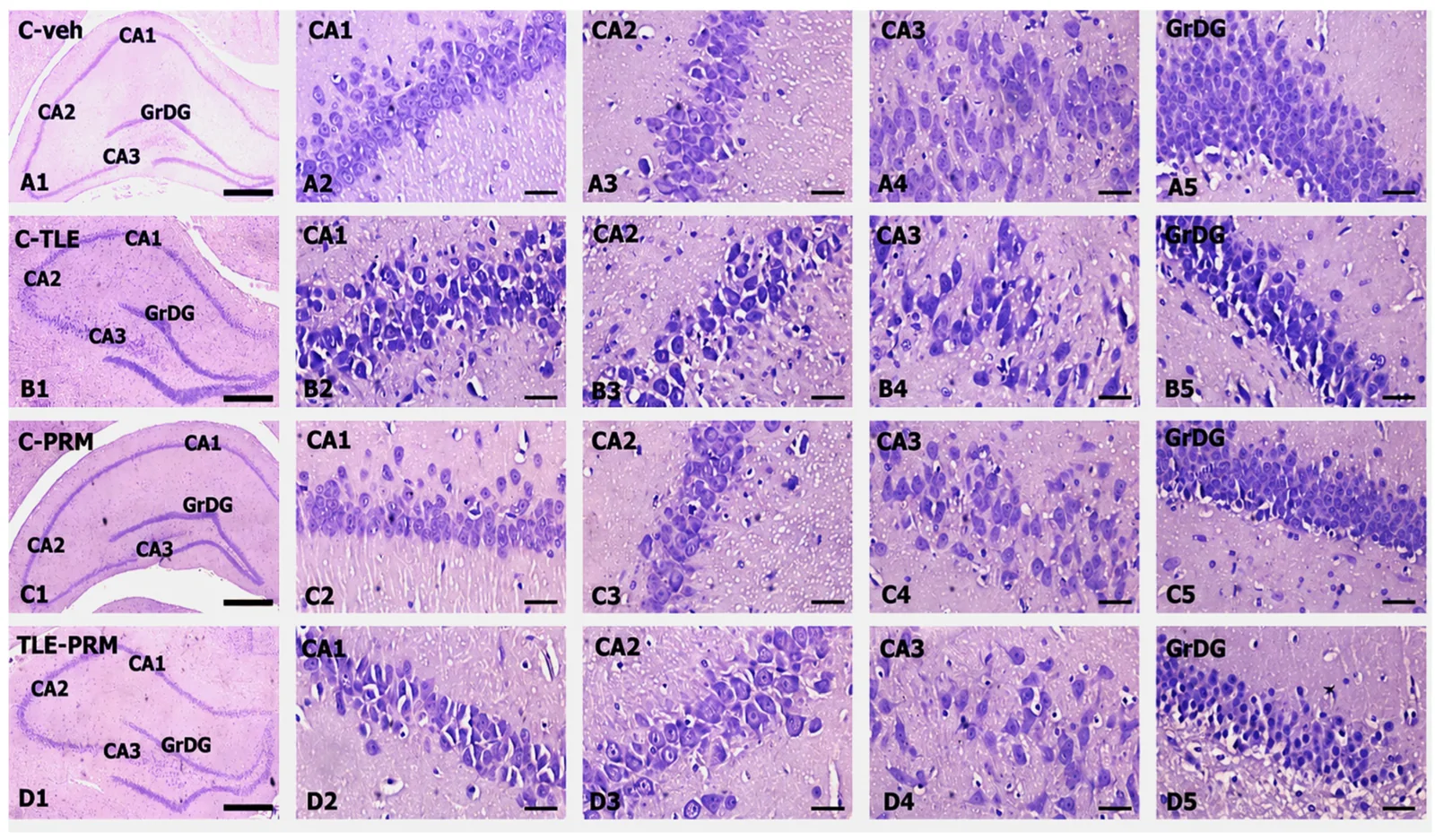
(**A**–**D**). Immunohistochemical expression of Neurokinin-B in the dorsal hippocampus. C-veh group (**A1**–**A5**), C-TLE group (**B1**–**B5**), C-PRM group (**C1**–**C5**), TLE-PRM group (**D1**–**D5**). Higher magnifications of the rectangles in all four groups are given. Scale bars = 200 μm (**A1**,**B1**,**C1**,**D1**); 40 μm (**A2**–**A5**,**B2**–**B5**,**C2**–**C5**,**D2**–**D5**).

**Figure 11:**
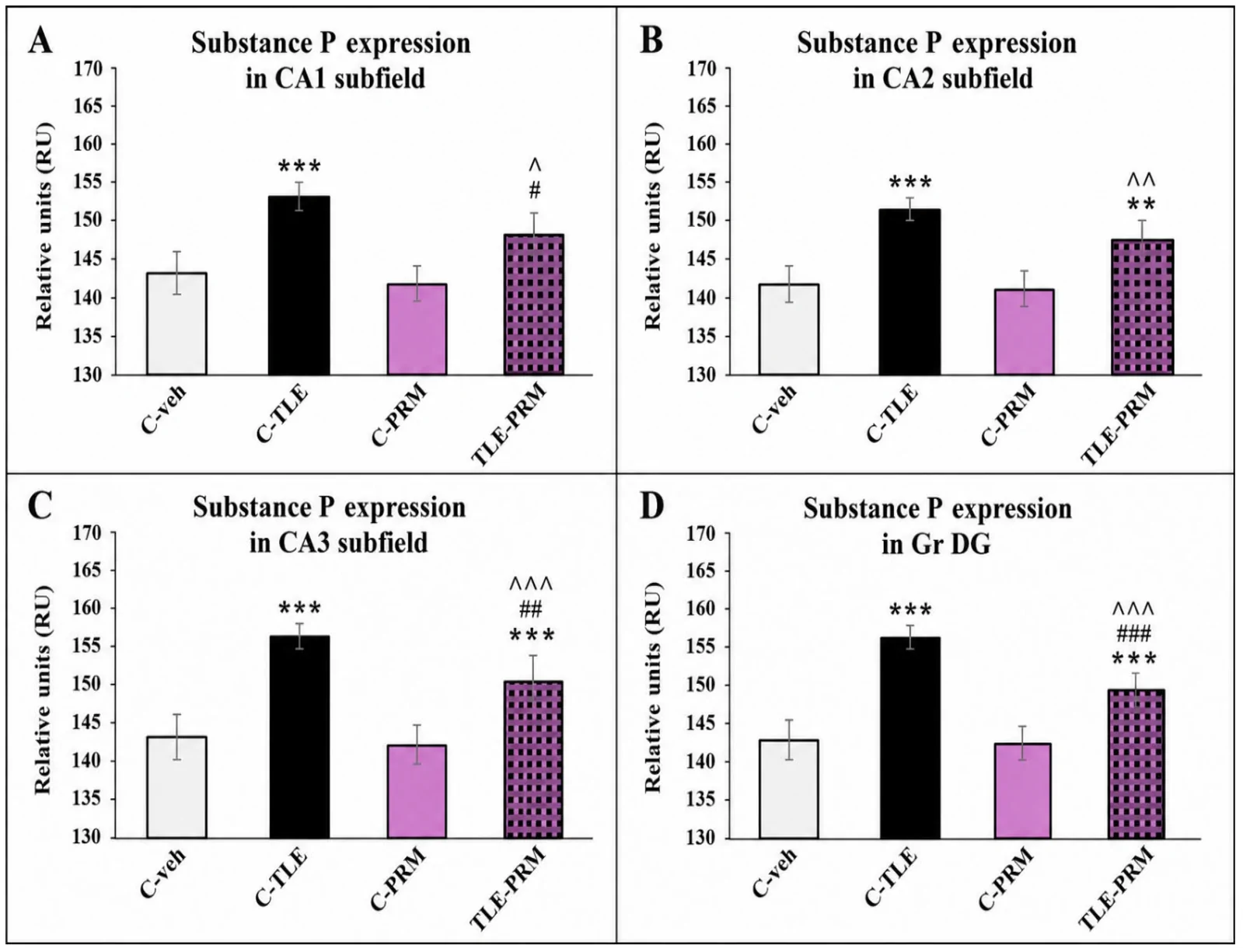
Substance P expression in the hippocampus of rats with lithium–pilocarpine-induced temporal lobe epilepsy and following perampanel (PRM) treatment. Quantitative analysis of Substance P immunoreactivity in the CA1 (**A**), CA2 (**B**), CA3 (**C**), and granular layer of the dentate gyrus (GrDG) (**D**). Substance P expression is presented as relative units (RUs) in the following experimental groups: vehicle-treated controls (C-veh), rats with temporal lobe epilepsy (C-TLE), perampanel-treated controls (C-PRM), and perampanel-treated rats with temporal lobe epilepsy (TLE-PRM). ** *p* < 0.01, *** *p* < 0.001 vs. C-veh; ^#^ *p* < 0.05, **^##^** *p* < 0.01, ^###^ *p* < 0.001 vs. C-TLE; ^ *p* < 0.05, ^^ *p* < 0.01, ^^^ *p* < 0.001 vs. C-PRM.

**Figure 12:**
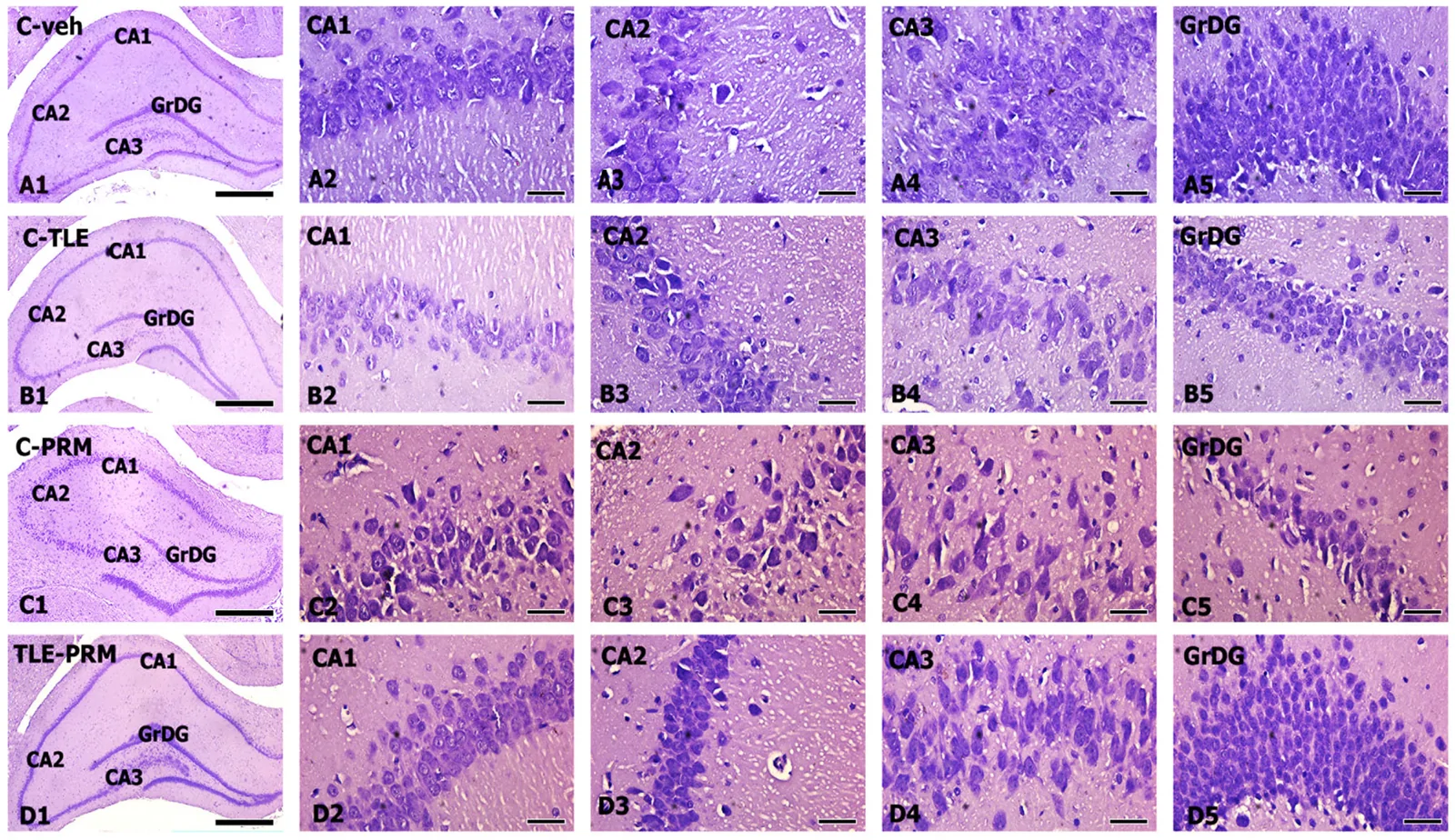
(**A**–**D**). Immunohistochemical expression of Substance P in the dorsal hippocampus. C-veh group (**A1**–**A5**), C-TLE group (**B1**–**B5**), C-PRM group (**C1**–**C5**), TLE-PRM group (**D1**–**D5**). Higher magnifications of the rectangles in all four groups are given. Scale bars = 200 μm (**A1**,**B1**,**C1**,**D1**); 40 μm (**A2**–**A5**,**B2**–**B5**,**C2**–**C5**,**D2**–**D5**).

## Data Availability

The data presented in this study are available on request from the corresponding author.

## Abbreviations

The following abbreviations are used in this manuscript:

PRM	Perampanel
TLE	Temporal lobe epilepsy
NKB	Neurokinin B
SP	Substance P
AEMs	Antiepileptic medications
NK1R	Neurokinin-1 receptor
NK3R	Neurokinin-3 receptor
